# The Exploitation of Toxic Fish from the Terminal Pleistocene in Maritime Southeast Asia: A Case Study from the Mindoro Archaeological Sites, Philippines

**DOI:** 10.3390/ani13132113

**Published:** 2023-06-26

**Authors:** Clara Boulanger, Alfred Pawlik, Sue O’Connor, Anne-Marie Sémah, Marian C. Reyes, Thomas Ingicco

**Affiliations:** 1 UMR 7194 Histoire Naturelle de l’Homme Préhistorique, Muséum National d’Histoire Naturelle, 75005 Paris, France; 2 Archaeology and Natural History, School of Culture, History and Language, College of Asia and the Pacific, Australian National University, Canberra, ACT 2601, Australia; 3 Japan Society for the Promotion of Science International Research Fellow, Department of Modern Society and Civilization, National Museum of Ethnology, Osaka 565-8511, Japan; 4 Department of Sociology and Anthropology, School of Social Sciences, Ateneo de Manila University, Quezon City 1108, Philippines; 5 TRACES ASIA, 3F Eduardo J. Aboitiz Sandbox Zone, Areté, Ateneo de Manila University, Quezon City 1108, Philippines; 6 Department of Early Prehistory and Quaternary Ecology, Eberhard Karls Universität Tübingen, Schloss Hohentübingen, 72074 Tübingen, Germany; 7 ARC Centre of Excellence for Australian Biodiversity and Heritage, Australian National University, Canberra, ACT 2601, Australia; 8 The National Museum of the Philippines, Manila 1000, Philippines; 9 School of Archaeology, University of the Philippines Diliman, Quezon City 1101, Philippines

**Keywords:** hunter-gatherer, marine environment, Diodontidae, poison, Indo-Pacific

## Abstract

**Simple Summary:**

Seascapes were the last environments to be discovered and mastered in the history of humankind. The adaptation to such environments therefore required the development of techniques considered as part of a set of distinctive innovations encapsulated in the concept of “modern behaviour”. This paper highlights the repeated catch and exploitation of toxic fish (e.g., Diodontidae, also known as porcupinefish in the Philippines), starting in the terminal Pleistocene ca. 13,000 years ago. The Bubog and Bilat archaeological sites yielded both cranial bones and dermal spines demonstrating that no preparation of the fish occurred immediately after the catch but rather that the fish were brought back to the camps. If not used for poison, these fishes would at least document the advanced knowledge to separate the toxic principle from the edible parts, which also implies a multi-stage cognitive process. Such knowledge was certainly one of the first steps toward the use of poison, meaning separating the edible parts from the toxic ones and keeping the latter for further use.

**Abstract:**

Representatives of the Diodontidae family (porcupinefish) are known to have been fished by prehistoric Indo-Pacific populations; however, the antiquity of the use of this family is thus far unknown. We report here on the presence of Diodontidae in the archaeological sites of Bubog I, II, and Bilat in Mindoro, Philippines, dating back to c. 13,000 BP (Before Present). This evidence demonstrates the early exploitation by islanders of poisonous fish. Every part of porcupinefish can be toxic, but the toxicity is mostly concentrated in some organs, while other parts are edible. The continuous presence of Diodontidae remains throughout the stratigraphic record of these Philippines shell middens suggests that porcupinefish were prepared by human inhabitants of the sites to render them safe for consumption, indicating an advanced cultural knowledge of the preparation needed to separate the toxic principle from the edible parts. This constitutes one of the rare examples of poison processing by humans, aside from the contentious wooden stick poison applicator from Border Cave (South Africa).

## 1. Introduction

The use of poison is culturally shared by all historically known hunter-gatherer groups [1,2], suggesting either that this behavior was a convergence acquired independently by the different groups, or that it is a cultural trait deeply rooted in time, with origins predating at least the later out-of-Africa dispersal by *Homo sapiens* [3]. The use of poison represents a major step in the evolution of cognitive skills, as it relies on advanced multi-stage processes in terms of techniques [1,4,5,6] and on the concept of deceit in terms of subsistence strategies and cultural adaptation to the environment [1].

Seascapes were the last environments to be discovered and mastered in the history of humankind [7]. The adaptation to coastal environments and the exploitation of aquatic resources forced humans to face never-before-encountered ichthyosarcotoxism amongst other challenges. The site of Madjedbebe on the Sahul Shelf, dated to c. 65,000 years BP, currently provides the earliest worldwide evidence for deliberate seafaring by our species [8,9]. Seafaring and intensive maritime use required the development of knowledge and technology considered an essential part of a new set of innovations distinctive of a “modern behavior” [10,11,12]. This theory of modern behavior, defining the why and the when of the emergence of modernity, was based on a series of traits, also sometimes glossed as a “package” of cultural innovations [5,13,14]. These new cognitive capacities were particularly useful, enabling adaptation to new environments often considered as impoverished or difficult to utilize, such as tropical rainforest [15] and coastal insular environments with sparse terrestrial fauna that require high-level or specialized technology to effectively exploit, such as composite hunting weapons and fishing gear [10,12,16,17,18].

Within this package of cultural innovation developed by *H. sapiens*, d’Errico and coauthors [5] reported a fragmented wooden stick from Border Cave, South Africa, which they interpreted, based on residue analysis, to be an applicator of poisonous ricin extracted from castor beans. This Later Stone Age tool, deriving from a layer dated to 24,000 BP, constitutes the earliest evidence for the use of poison by humans. However, the interpretation of the function of this artifact, as well as the toxicity of the ricin, have been challenged by Evans [6]. Regardless of whether or not it was used for the application of poison, this tool at least documents an advanced knowledge of the preparation required to separate the toxic principle from the edible parts of the castor beans [19], which still demonstrates a multi-stage cognitive process never before reported. This highly advanced knowledge of poisons would have been acquired by steps, initially through the identification of the toxic part of a plant or animal.

**Figure 1:**
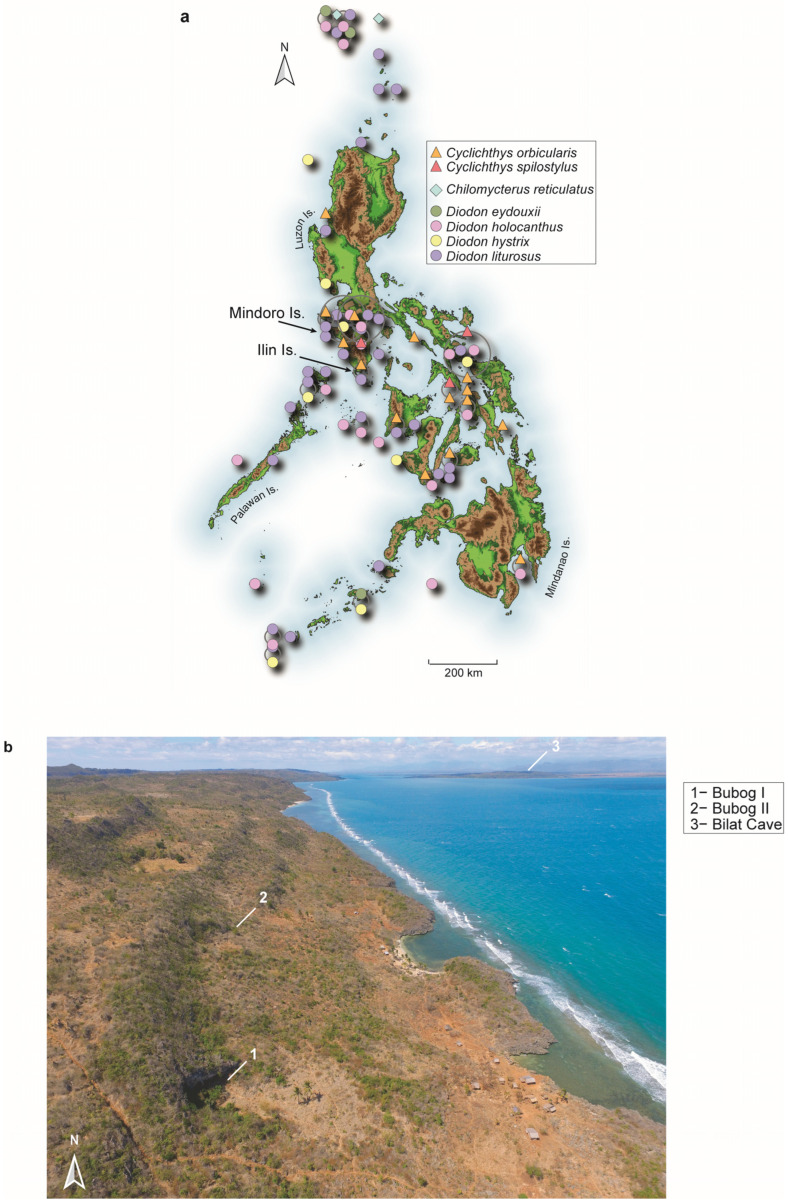
(**a**) Taxa diversity surrounding the Philippine islands (transformed data from fishbase.org, accessed on 1 May 2023) and location of Mindoro and Ilin islands where the archaeological sites are situated. (**b**) Aerial view of Ilin Island and Ilin Strait and location of the three archaeological sites. Bubog II is located about 300 m north of Bubog I; Ilin Island is currently less than 1 km off the west coast of Mindoro and Bilat Cave is situated approximately 8 km north of the Bubog sites.

**Figure 2:**
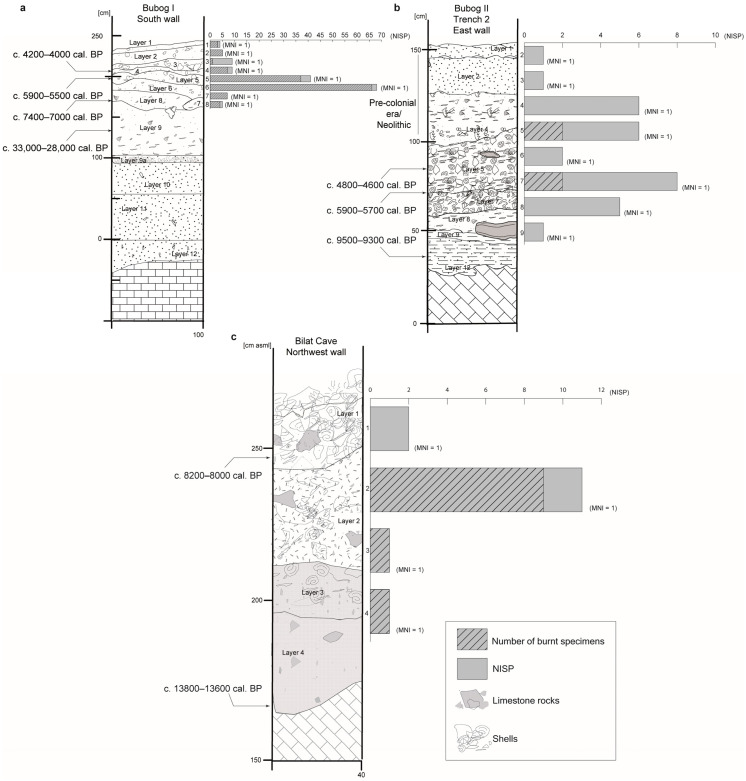
Stratigraphic profiles and Diodontidae Number of Identified Specimens (NISP) and Minimum Number of Individual (MNI) per layer at (**a**) Bubog I, (**b**) Bubog II, and (**c**) Bilat Cave and corresponding Number Identified Specimens (NISP) of Diodontidae for every layer. The hatchings represent the number of burnt specimens for each layer. Radiocarbon dates cited were calibrated with OxCal 4.4 (using the most recent calibration curves of IntCal20 (file version intcal20.14c) for dating charcoal, and Marine 20 (file version marine20.14c) for dating marine shells and reported as modeled data at 95.4% confidence intervals) [21,22,23]. Dates published before 2020 were corrected accordingly where necessary.

Here, we report on a similar behavior: the repeated exploitation of toxic fish in the rockshelters of Bubog I and Bubog II, and in Bilat Cave, southern Occidental Mindoro, Philippines [20] (Figure 1), whose occupation deposits date from the terminal Pleistocene to the early/mid-Holocene and modern era (Figure 2).

## 2. Materials and Methods

### 2.1. Study Sites

Bubog I is a rock shelter on the eastern coast of Ilin Island at c. 30 m AMSL (Figure 1). It has produced a human-derived stratified shell midden dating from c. 35,000 to 4000 cal. BP, beneath which are terrestrial silty deposits with some cultural remains that currently remain undated [24,25,26] (Figure 2). Fish remains belonging to 19 families were found throughout all layers (NISP = 1445) [25,27,28]. The Late Pleistocene occupants of Bubog I successfully exploited a large range of inshore marine resources, as well as inland resources, with a notable shift from mangrove swamps to open reef from Layer 8, dating to around 7500 years ago [24,25,27]. Among the larger terrestrial taxa recovered were wild native pig, *Sus oliveri*, two endemic deer species, *Rusa marianna* and *Cervus alfredi*, and the tamaraw, the endemic dwarf Mindoro buffalo *Bubalus mindorensis* [20,25,27], all currently absent on Ilin Island, but the vast majority of the mammalian remains recovered belong to the recently extinct endemic giant cloud rat *Crateromys paulus* [29]. The two lowermost layers contained sparse mollusk assemblages, unlike the higher stratigraphic units. Layers 9 and 8 were rich in mangrove shells such as *Terebralia sulcata*, as well as the mud crabs *Cardisoma carnifex* and *Scylla serrata* [25,30]. In the upper layers, beginning with Layer 7, marine intertidal shells such as *Nerita undata* are abundant. Evidence for the use of marine shells as raw material for tool making dates to as early as 33–28,000 cal. BP with several flaked *Geloina coaxans* valves and one *Tridacna* shell edge-ground adze blade that was directly dated to 7347–7016 cal. BP [20,24,31]. A few small fragments of flaked obsidian were also recovered from the base of the shell midden and the immediate underlying layer, evidencing early inter-island networking dating back to c. 33,000 BP [26,32].

Bubog II is a rock shelter located about 300 m north of Bubog I at a slightly higher elevation of 45 m AMSL (Figure 1). Like Bubog I, it is close to the shore, from which both sites can currently be reached by a 10 min walk. The site is comprised of a shell midden showing a similar structure to its neighbor, although less extensive, with a stratigraphic profile of 14 distinct layers in the eastern Trench 2, spanning from c. 9500 cal. BP to 16th–17th century AD (Figure 2). The earliest date thus far obtained for Bubog II is 11,061–10,522 cal. BP on associated charcoal, retrieved from Trench 3 in the center of the habitation floor. The faunal and lithic assemblages, although less substantial, are similar to Bubog I [26]. Fish remains belonging to 19 families were found throughout all layers (NISP = 1382) [28]. An unfinished *Tridacna* preform retrieved from Trench 3 was dated to 8971–8600 cal. BP on associated charcoal [26].

The third site, Bilat, is a cave at a maximum elevation of just 3 m AMSL, located in Santa Teresa on the southern coast of the main island of Mindoro (Figure 1). It has three openings, one facing the land side, the other two the narrow Ilin Strait and Ilin Island. Ilin Island is currently less than 1 km off the west coast of Mindoro and Bilat Cave is situated approximately 8 km north of the Bubog sites which are visible from there. Being located close to current sea level, Bilat Cave was probably flooded during the Holocene climatic optimum around c. 6000 BP when the sea level was c. 3–5 m higher than at present [33,34]. A large shell midden covered the surface of the entrance area, and two excavation trenches, including a shell-sampling square, were opened here. A total of four layers were identified with a radiocarbon chronology ranging from 20,830–20,310 cal. BP in the main trench, to 13,790–13,596 cal. BP until present day (287–3 cal. BP) within the shell midden where most of the fish remains were recovered (Figure 2). Parts of the cave are still used by modern fishers for storage, and two of the cave’s three entrances open directly onto the Ilin Strait [20,26]. The excavation produced fragmented remains from terrestrial and marine vertebrates and some lithic artifacts. Fish remains belonging to 15 families were found throughout all layers (NISP = 678) [28]. Pawlik and Piper [26] also reported the finding of an edge-ground *Tridacna* shell adze dated to 7291–6981 cal. BP, which is similar in form and age to the one found in Bubog I [31]. The similarities between these artifacts suggest a relationship to the Bubog sites across the Ilin Strait and the widespread use of this particular type of tool technology during the early to mid-Holocene [26,35].

All three sites yielded quite diversified fish remains with no less than 19 families reported at Bubog I and Bubog II and 14 at Bilat Cave [28]. Most of the specimens were probably taken along the shore within the coral reef zone in different kinds of substrates (from rocky to sandy areas) or in the mangrove swamps, using line fishing and nets/traps [25]. The recorded fish taxa ranged from Carcharhinidae (requiem shark) to Scaridae (parrotfish) and Balistidae (triggerfish), to name just a few, but notably also included the toxic Diodontidae (porcupinefish), which we discuss here.

### 2.2. Taxonomy

The Tetraodontoidei suborder (Diodontidae and Tetraodontidae) and, more generally, Tetraodontiformes are highly derived teleosts (bony fishes) with extremely modified skin appendages and exhibit morphological diversification both in terms of their craniofacial and dermal skeletons [36]. As a defense mechanism, their spines erect, and their body inflates and eventually swells like a balloon because of their ability to ingest water or air into gas bladders, their absence of ribs, and the extreme elasticity of their ventral and lateral skin [37,38].

Currently seven species of Diodontidae are known along the Philippine coasts. Two of them belong to the genus *Cyclichthys* (*C. orbicularis*, *C. spilostylus*), one to the genus *Chilomycterus* (*C. reticulatus*), and four to the genus *Diodon* (*D. eydouxii*, *D. holocanthus*, *D. hystrix*, *D. liturosus*) (Figure 2), while the family Tetraodontidae is far more diversified with 9 genera and 34 species found in the area [39].

### 2.3. Ecology of Tetraodontoidei Fishes

**Figure 3:**
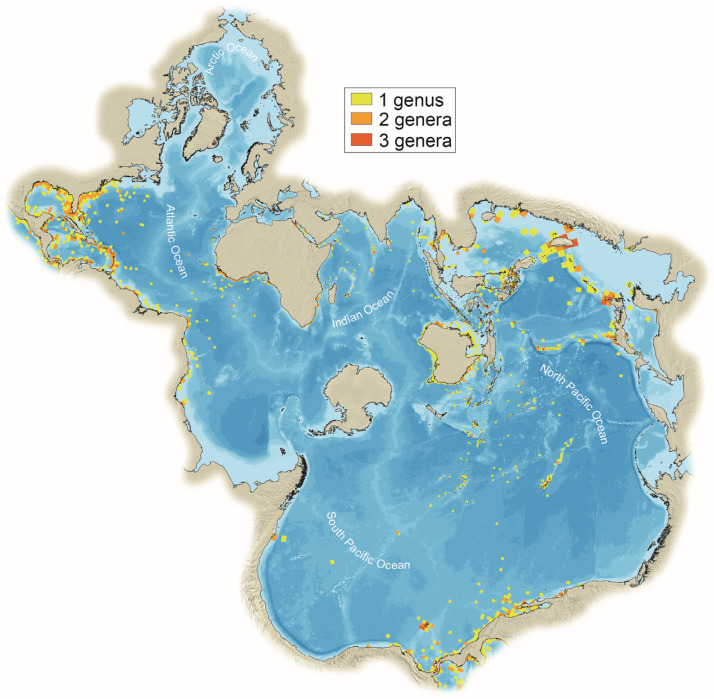
Worldwide richness distribution (isometric 1 × 1 degree squares) of Diodontidae in Spilhaus projection (transformed data from fishbase.org, accessed on 1 May 2023).

Diodontidae, commonly referred to as porcupinefish, as well as its close relative Tetraodontidae (pufferfish) are distributed worldwide, although they are most common in Asia [39,40] (Figure 3). They have a wide diversity of diets, from invertivorous to herbivorous, omnivorous, and even carnivorous [38], and inhabit marine environments from pelagic to coastal areas but can also be found in fresh waters [39,41]. However, most of the members of this fish suborder favor shallow sandy bottoms and reefal areas [42].

### 2.4. Zooarchaeology

**Figure 4:**
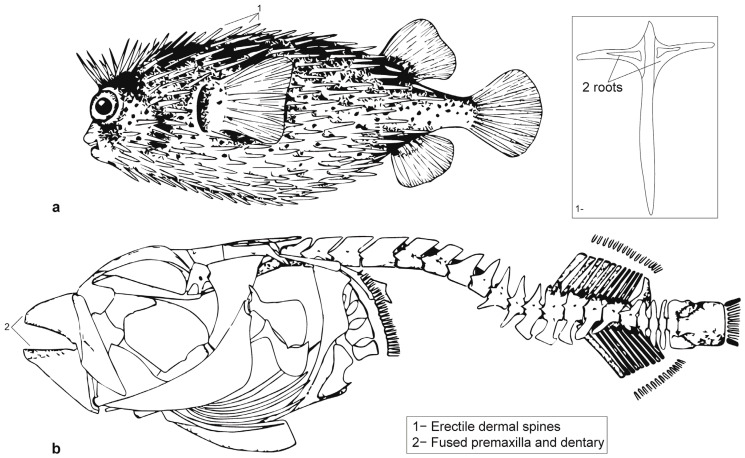
(**a**) *D. holocanthus* (longspined porcupinefish) (max. TL = 50.0 cm) with (**b**) dermal spines of *Diodon* sp., c. skeleton of *D. holocanthus* after Carpenter and Niem [46] and Tyler [51]. Source (**b**): Food and Agriculture Organization of the United Nations. Reproduced with permission.

**Figure 5:**
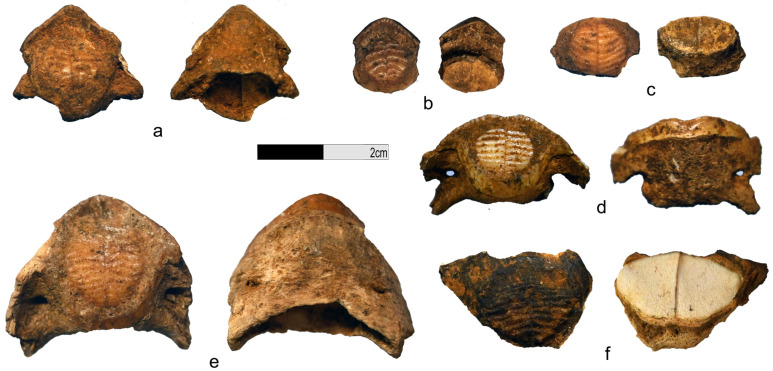
*Diodon hystrix* (**a**) premaxilla, Bubog II, Layer 5; (**b**) dentary, Bubog I, Layer 1; (**c**) dentary, Bilat Cave, Layer 2; (**d**) dentary, Bubog II, Layer 7; (**e**) premaxilla, Bilat Cave, Layer 2; (**f**) premaxilla, Bubog I, Layer 5.

Representatives of the Diodontidae present some osteological characteristics that make their identification straightforward in archaeological records [43,44,45]. To begin with, their body is covered with numerous dermal spines (200 to 500 spines per individual depending on the species and specimen) that are easily observable to the naked eye [43]. Tetraodontidae’s dermal spines, being smaller and sometimes even microscopic, are not recorded in the archaeological record. Diodontidae and, more particularly, *Diodon* sp. dermal spines usually have a characteristic oval cross-section and two movable roots [43,46] (Figure 4). While easily identified, this element can cause issues in overestimation and is by no means suitable for estimating the minimum number of individuals (MNI) [47,48,49,50]. Diodontidae’s jawbones are also easily identifiable as both dentary and articular, and premaxilla and maxilla are fused [51] (Figure 4 and Figure 5).

In the present study, the Diodontidae remains come from the excavated sediments of the three sites that were sieved through a 2 mm mesh screen and bagged for flotation at the beach, then sieved again with a 0.5 mm mesh. All fish remains were sorted from the assemblage [24]. The Diodontidae specimens have then been identified utilizing the osteoichthyological reference collection of the *Muséum national d’histoire naturelle* in Paris (UMR 7209 “*Archéozoologie et Archéobotanique—Sociétés, Pratiques et Environnements*”) in which three representatives of the Diodontidae family, (*Diodon holocanthus* (No. 00141), *Diodon hystrix* (No. 00211), and *Cyclichthys orbicularis* (No. 00224), are housed. For each site, the number of identified specimens (NISP), as well as the minimum number of individuals (MNI) and the minimum number of elements (MNE), have been calculated. The MNI has been calculated using the occurrence of jaw bones (premaxilla and dentary), or dermal spines, when jaw bones were absent, the presence of one dermal spine or more counting for only one individual. The external surfaces of the remains were assessed for the presence of anthropogenic bone modifications (e.g., cut marks) using a hand lens (x10). Black, grey, and brown burning coloration [52,53] were identified from their homogeneous shapes with gradual and often partial coloration as a result of localized heating points (as opposed to dendritic-shaped manganese stains). Upon completion of the project and subsequent analyses, all materials will be returned to the National Museum of the Philippines as the country’s repository for cultural property.

## 3. Results

In total, we counted 193 Diodontidae remains from the three sites in Occidental Mindoro.

### 3.1. Bubog I

**Figure 6:**
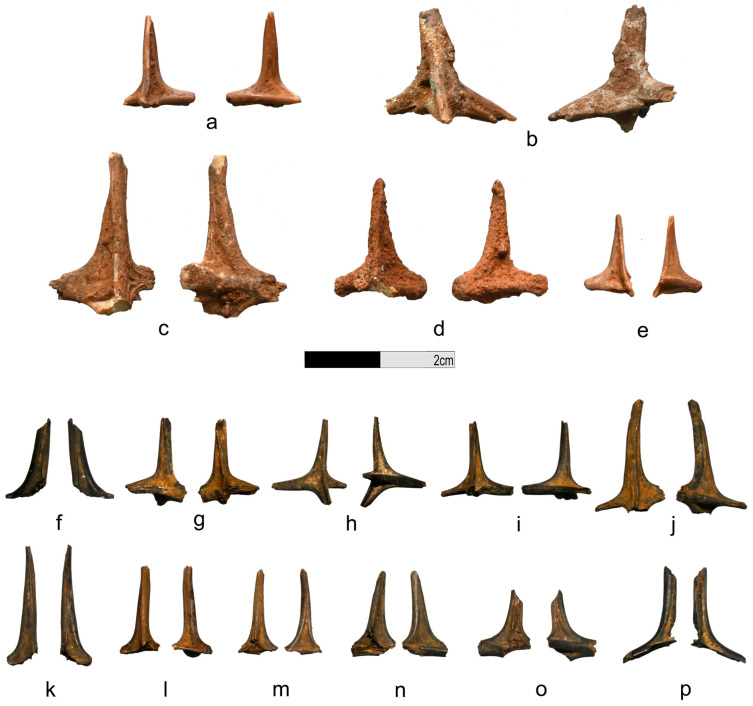
Diodontidae dermal spines, (**a**) Bubog I, Layer 2; (**b**) Bubog I, Layer 5; (**c**) Bubog II, Layer 4; (**d**) Bubog II, Layer 5; (**e**) Bubog II, Layer 8; (**f**–**j**) Bubog I, charred dermal spines, Layer 5; (**k**–**p**) Bubog I, charred dermal spines, Layer 6.

Among the 19 families and the 1445 fish remains identified at Bubog I, the Diodontidae are dominant in terms of number of identified specimens (NISP = 148) [28]. They have been identified from Layer 8 to 1 (c. 7400 cal. BP until after 4000 cal. BP) with two peaks, in Layer 6 (NISP = 68) and 5 (NISP = 41); the MNI is 7 (Figure 2). Most were dermal spines (MNE = 137) (Figure 6). A dentary was identified in Layer 1, as well as one premaxilla and an unidentified jaw fragment in Layer 5 (5628–5309 cal. BP), all belonging to the species *D. hystrix* (spot-fin porcupinefish) (Figure 5). Burning marks were present on 130 of the bones (Figure 5 and Figure 6) representing 12.52% of the burnt remains found at the site (NISP = 1038) (Supplementary Table S1).

### 3.2. Bubog II

We recovered 30 Diodontidae remains at Bubog II, from Layers 9 to 2 (early Holocene to pre-Spanish era), among the 19 fish families and 1382 remains identified at the site. They are the third dominant taxon at the site, after the Scaridae (parrotfishes) and the Balistidae (triggerfishes) [28]. A total of 28 Diodontidae dermal spines, as well as two jaw fragments (a dentary and a premaxilla) of *D. hystrix*, were recovered from Trench 2 associated with a date of 5927–5747 cal. BP, where the NISP is the highest (NISP = 8), and from Layer 5 in Trench 3, dated to 4868–4653 cal. BP (Figure 5 and Figure 6). The MNI is 8 (Figure 2). Four bones (13.3%) exhibited burning marks, which is proportionally in agreement with the 13.46% of the whole 1382-bone assemblage of the site that exhibits burning coloration [28] (Figure 5 and Figure 6; Supplementary Table S2).

### 3.3. Bilat Cave

At Bilat Cave, 15 fragments of Diodontidae were recovered from Layers 4 to 1 (c. 13,000 BP to present) among the 678 fish remains and 14 families identified. They are the second dominant taxon at the site, after the Scaridae (parrotfishes) [28]. The MNI is 4 (Figure 2; Supplementary Table S3). Most of the Diodontidae remains are dermal spines (MNE = 12). A dentary and a premaxilla belonging to the species *D. hystrix* have been identified in Layer 2 and a Diodontidae dentary in Layer 3 (Figure 4). Diodontidae is the second most dominant taxa, after the Scaridae (parrotfish), at the site [28]. Eleven of the identified fragments (73%) have been partially or totally burnt, conforming to 80.68% of fish specimens recovered at Bilat Cave that presented burning marks [28] (Figure 2; Supplementary Table S3).

## 4. Discussion

The investigated Ilin Strait sites are mainly composed of marine taxa [24,25,28,30]. Fish, and particularly porcupinefish, were exploited throughout all cultural layers, from the end of the Pleistocene through to the beginning of the Spanish colonial era. They are predominant at Bubog I (NISP = 148), notably in Layers 6 (NISP = 68) and 5 (NISP = 41), dated to the mid-Holocene. At Bubog II (NISP = 30), Layers 8 to 5, dated to around the same period, yielded most of the remains (NISP = 21). At Bilat Cave (NISP = 15), located on the coast of Mindoro where environmental settings were and are slightly different from the two other sites, most of the remains were associated to Layer 2, dated to the early Holocene, while only two remains were recovered from Layer 1. While, as previously discussed, the NISP can cause overestimation issues, the low MNI of Diodontidae has the contrary effect. It is not representative of the actual number of individuals that were brought back to the sites, as most of the remains identified were dermal spines—furthermore, different sizes of spines were observed during the identification process, meaning that there are more individuals per layer than are reflected in the MNI (Figure 2).

The context of the finds, the broken shells, and the burning marks on several bones are evidence for the human derivation of these accumulations. No marks such as, for instance, digestive or gnawing marks, of any other biotic agent have been identified. No anthropogenic cut marks were identified on any of the Diodontidae remains, but experiments have shown that cut marks preserve poorly on fish bones once buried [54]. All the remains were fragmented, which added to the difficulty of identifying them to genus or species level. The recovery of a polished bone fishing gorge used as bait and modified pebbles used as net sinkers at Bubog I are additional evidence for the anthropogenic accumulation of the midden and fish bones and are further indications of the use of a variety of fishing techniques [25]. Yet, one cannot discard the possibility that the porcupinefish might have been gleaned along the shore when floating while inflated, therefore requiring no specialist technique or equipment for their capture [47,55]. We observed such a catch by the present-day inhabitants of Ilin Island, and a similar behavior has been reported to us from Samar Island, on the east coast of the Philippines (J. Magloyuan, personal communication, 6 December 2022).

Bubog I and Bubog II are today located at about 30 m and 45 m, respectively, above msl and c.180 m in a straight-line distance from the coast (Figure 1). The porcupinefish were, therefore, brought back to cave campsites along a partially steep trail, rather than being discarded immediately back into the sea, which would be the expected scenario if they were not of any interest to the fishers. Whether the Diodontidae were carried to the sites incidentally, within a net along with other fish intended for consumption, is uncertain. In such a case, the toxic porcupinefish could have been discarded at the camp only when the content of the net was checked. However, based on the study of Brainerd [37] on the comparative stomach volumes of a *D. holocanthus* at rest and when water-inflated, one can estimate that a water-inflated *D. holocanthus*, which ranges between 40 to 90 cm long, would weigh between 900 g and 2.2 kg, which would represent a certain energy cost in its transport from the shore up to the Bubog rock shelters. This extra carrying cost seems unnecessary if the fish were simply discarded after returning to the camp. No data at hand allows us to make a similar estimate for an inflated *D. hystrix*, but the weight would have probably been proportionally about the same. At rest, *D. hystrix* can weigh up to 2.8 kg [39], which would become quite heavy when inflated. Furthermore, the proportion of burnt Diodontidae remains—145 spines of Diodontidae were totally or partially burnt—at each site is similar to the proportion of burnt specimens observed for the other fish bones, which indicates these fish were not processed to remove the toxins at the shoreline but rather were transported whole back to the sites. We also believe that the Diodontidae may have been brought to the camp separately from the other fish catch since, if collected and transported as a mixed catch and then discarded, there would have been a high risk of spoiling the rest of the catch they were transported with, as the skin of the Diodontidae can secrete the toxin (Table 1). This effort to bring poisonous fishes from the shore to the rock shelters was observed in almost every cultural layer of the three Ilin Strait sites. The clear anthropogenic origin of all the fish remains recovered, the continuous presence of Diodontidae in the cultural layers, the effort that carrying these specimens from the shore to the sites would have required, and the repeated burning of the spines, leaves little doubt that Diodontidae were intentionally caught and returned to the shelters.

Most marine and freshwater Diodontidae and Tetraodontidae (pufferfish) are toxic [38,43,58,59,60]. This toxicity is primarily caused by tetrodotoxin (TTX) or to a lesser extent by saxitoxin (STX) or other analogs such as anhydrotetrodotoxin [56,61]. In freshwater puffers, this toxin is partly synthesized [62], while it mostly results in porcupinefish from the accumulation of TTX-producing bacteria—either through the food chain or parasitism—present in the environment to which porcupinefish, puffers, and some other marine animals are tolerant [56]. It usually results from the fact that the liver first uptakes TTX, which is then accumulated in other organs or muscles [61] (Table 1). Some species of porcupinefish that have been previously documented as nontoxic can still accumulate large amounts of TTX. For instance, while *D. hystrix* has an acceptable level of toxicity in Okinawa, Japan [57] (Table 1) and is considered a delicacy [40], its consumption has become taboo in Fiji where numerous cases of food poisoning have been recorded over time, notably for women during pregnancy or breastfeeding [63,64]. Although the concentration of the toxin in the different parts of the fish has been proven to vary during the year in most species [43,57,59,65,66,67], there is no such thing as a ‘safe season’ for their consumption [68]. The presence of TTX in porcupine and pufferfish primarily depends on the presence of TTX-producing bacteria. Twenty-five bacteria genera have been reported to produce TTX with *Vibrio*, *Aeromonas*, and *Bacillus* the most common [69]. These bacteria are all ubiquitous and present worldwide [70]. *V. alginolyticus* has been identified as the main TTX producer [69]. In the Philippines, this bacterium, which causes vibriosis in fish, has been documented at several locations [71,72,73,74], evidencing that TTX- and STX-producing bacteria are present in the seas surrounding the archipelago. Additionally, the local fishers in Occidental Mindoro say that “butbutan” (Diodontidae) and “butiti” (Tetraodontidae) are poisonous. Any by-catch of one of these taxa today would be disposed of immediately (H. Yap, M. Webb, personal communication, 26 February 2022).

Environmental factors further seem to play an important role in the level of toxicity of each species and even individuals [69]. TTX is in higher amounts in fish during the spawning season—currently between spring and summer, probably because the fish use TTX to protect their eggs from predators [75]—making them more dangerous to eat at this time of the year [40]. The concentration of TTX in the fish body will also depend on season and age [66,76]. Because the level of toxicity of porcupine and pufferfish is environmentally dependent, it has been suspected that the concentration of TTX in Diodontidae and Tetraodontidae could be higher with higher temperatures and at higher sea-level stands [61,77], a condition partly met on Ilin Island when early fishers were bringing these fish back to the Bubog rock shelters and Bilat Cave [24], between 7000 to 4000 BP during the Holocene climatic optimum, when the sea level was at its highest [78]. However, this must be treated circumspectly, as while *Vibrio* development is strongly correlated with high temperatures [79], at present there is no reason to suspect that the concentration of TTX 13,000 years ago in the Philippines when the first consumption of toxic fish is first registered was different from the present conditions.

It has been demonstrated that puffers can identify tetrodotoxin in the environment by their sense of smell [80], which shows that this toxin has a special chemically odorant signature. Yet, there is no evidence at present that any animal other than puffers themselves who are TTX bearers can detect this toxin by smell. It is furthermore very unlikely that microsmatic *H. sapiens* would be capable of doing so, whatever the olfaction skills of hunter-gatherers. Because of the capacity of porcupine and pufferfish to bioconcentrate large quantities of TTX, they become highly toxic and lethal to humans and predators in general [75]. In humans, symptoms of TTX ingestion usually appear three to 30 min after eating [76,81]. First observed clinical manifestations are perioral paresthesia, weakness of the two lower limbs, headache, difficulty in breathing, nausea and vomiting, blurring of vision, vertigo, dizziness, and cramping pain in the lower limbs, which rapidly lead to ascending paralysis, coma, and death within 24 h for 50 to 60% of the persons who ingested it [40,57,75,76,81]. To be eaten, the viscera, which are the most toxic parts, should be removed as quickly as possible [68] (Table 1). Captain Cook and his HMS Resolution crew suffered in 1774 from TTX poisoning after consuming a puffer’s liver and roe while anchoring off an island in New Caledonia, and his record provides the earliest description of the symptoms related to such fish poisoning [82]. Today, the lack of data for many species and areas [57] and the ignorance regarding their proper cooking [40] makes the consumption of porcupine and pufferfish controversial.

Coastal ethnic communities around the world largely rely on seafood consumption [83], and so did the earliest settlers of the oceanic islands of the Indo-Pacific [25,28,84,85]. Tetraodontoidei poisoning is most common in Asian coastal populations where it is the main cause of death by food [86]. Although the majority of the studies focusing on Tetraodontiformes’ toxicity were conducted on Japanese specimens (Table 1)—because “fugu” are one of the finest delicacies in the Archipelago of the Rising Sun—most of the cases of poisoning by puffer consumption were reported from countries surrounding the South China Sea [65,87,88,89,90,91,92]. In the Philippines, in 2022, one death and seven injured people were reported from Cebu [93]. In 2020, three deaths were reported from the Visayas, while four other persons who ate at the same barbecue stand recovered at the hospital [94]. Another case from 2019 reports on 16 persons recovering at the hospital from puffer poisoning at Ozamiz City, Mindanao, while another one was more seriously affected [95]. In the archaeological sites of Ilin Strait, one single *H. sapiens* individual has been recovered at Bubog I [96]. There is therefore no evidence for any correlation between the continuous and common occurrence of poisonous fishes and any accumulation of human remains.

In several communities around the world, porcupine and pufferfish are regarded as taboo. This is the case in Brazil where puffers are tabooed all year long [97,98,99], as well as in Fiji [63,64]. However, in other parts of the Indo-Pacific, porcupine and pufferfish are carefully prepared to remove the toxic parts and then consumed. In Betong, Malaysian Borneo, a pluri-century festival is organized yearly—mostly for tourists nowadays—around the catch and consumption of large quantities of the *Lagocephalus lunaris* that gather in the Saribas River to lay their eggs [100]. Although this pufferfish is said by the locals to be safe to eat, it does contain TTX [59], which has resulted in some side effects following its ingestion [100]. From our own observations in Timor-Leste, whenever porcupine fish are caught—which often happens during low tide, sometimes with basket traps—the guts, the skin, and the dermal spines are removed prior to consumption [28]. Ethnographic studies also report that Malay populations used to take out the head and the guts of the fish [76], as ordinary cooking (heating/roasting or boiling) procedures do not destroy nor alter the toxicity of the fish and, thus, do not make it safe for consumption [81]. Only withstanding boiling for six to nine hours would modify the efficiency of the poison [76]. After removing the viscera, the pufferfish are boiled in water to remove the tinik (e.g., dermal spines), and the flesh is then removed for cooking (J. Magloyuan, personal communication, 6 December 2022). It appears from this ethnographic review that puffers are either taboo all year long, or considered edible only after the toxic parts are removed through a complex multi-stage preparation process. There is no in-between, meaning that puffers are never part-year taboo. Nor are they ever fully consumed without removing the most common toxic parts (Table 1). However, depending on the environment, some specimens may have a lower toxic level. In the Bubog and Bilat sites, the recovery of both cranial bones and dermal spines indicates that no preparation of the fish immediately following the catch occurred shoreside, but rather they were brought back to the camps with spines intact.

Diodontidae also appear to have been part of the diet of Pacific populations and have been documented in several Neolithic-aged zooarchaeological records at sites in Micronesia [101,102,103,104,105,106], Melanesia [47,63,107,108], and Polynesia [44,45,48,109,110,111,112]. Some of these sites recorded high proportions of Diodontidae remains and sometimes also Tetraodontidae remains, whereas, in Island Southeast Asia, only one instance of a cutmark on a Diodontidae dermal spine, associated with other remains of the same taxa, has been reported at the Neolithic archaeological site of Pamayan, Batanes Islands, Northern Philippines [49]. Ethnographic accounts on the Batanes Islands suggested to the authors that spines of porcupine fish were deliberately removed during butchery using sharp tools and buried in the sand [49]. Twenty remains of Diodontidae and the same amount of Tetraodontidae were recovered from the large Holocene assemblage (NISP over 20,000) of Asitau Kuru (Timor-Leste) [28,113]. Six remains of Diodontidae and four of Tetraodontidae were identified at Here Sorot Entapa (Kisar, Indonesia) [84], while 49 specimens of Diodontidae were counted at Tron Bon Lei (Alor, Indonesia) [85].

The findings from our archaeological data further highlight the social aspects of fishing [114]. The consumption, in the Philippines, of porcupinefish, over several thousand years in Ilin Strait, is a statement to the persistence of cultural practices across generations of established islanders, perhaps indicating a continuity in the occupation of the sites by the same cultural groups of people sharing the same knowledge [28]. It can be seen as the last step toward coastal adaptation with the selection, acquisition, preparation, and consumption of these fish for either the nutritional, technical, or social values that they carry. Additionally, once the poisonous parts have been identified and removed, these discarded parts could also be used specifically for their toxicity, for hunting, or as bait for fishing. Gimlette [76] reported that, in Kelantan, Malay populations mix the gall of two locally occurring fish species, *Tetraodon fluviatilis* and *Takifugu oblongus*, with other local toxins in order to create a powerful poison. Such a mixture could have been coated on hunting devices such as arrowheads [1] or traps used to catch small animals. A small injection of TTX into a prey can cause a fall in blood pressure, slowing of the circulation with cardiac tremor, fibrillation, and paralysis, followed by death [76]. At the Bubog sites, the high frequency of giant cloud rats *Crateromys paulus* has been interpreted as human-derived prey, but their method of capture remains unclear [30]. Whether they were hunted with some sort of poisoned darts from TTX extraction after pufferfish processing is unknown but not improbable.

TTX was also used by some tribes for performing rituals, as was the case at Tikal (Guatemala) by the Maya [115] and in Haiti [116,117,118,119]. One should further consider that the risk one takes when eating toxic food such as porcupinefish or pufferfish is in itself neurally stimulating, and the same may have been true for the early Mindoro settlers who might not have sought to remove the toxic parts before consumption but preferred to look for perilous quests to face [120].

## 5. Conclusions

In the case of the Bubog I and Bubog II rock shelters and Bilat Cave, Diodontidae were recovered from the assemblage and are not considered an accidental by-catch. The presence of burning marks on several of the Diodontidae spines shows the anthropogenic origin of the accumulation. This burning, however, was incapable of removing the toxin for safe consumption of the flesh. As of now, we have no evidence whether the Diodontidae were acquired for direct consumption or for other purposes, such as for using their poison for future hunts. The fact that their bones mirror the other fish bone in the cultural assemblage to which they were mixed in the archaeological layers has been interpreted, at the very least, as indicating an advanced knowledge of the preparation needed to separate the toxic principle from the edible parts, whether this was for consuming their flesh or for exploiting their toxin, which requires a multi-stage process. Such knowledge was certainly one of the first steps toward the use of poison, meaning separating the edible parts from the toxic ones and keeping the latter for further use. The processing and use of toxic fish represent the final step toward modernity related to coastal adaptation, and the data from the Ilin Strait sites constitute some of the earliest evidence of processing of poison, in addition to the challenged wooden stick poison applicator discovered at Border’s Cave in South Africa [5,6,19].

## Figures and Tables

**Table 1:** Edibility and toxicity of Japanese porcupine and pufferfish organs after Mastuura [43], Noguchi and Arakawa [56], and Khora [57]. For Khora [57], only the highest concentration of toxin have been taken into account. Taxa in bold letters: species present on the Philippines coasts after Froese and Pauly [39]. In light grey: edible parts; in dark grey: nonedible parts; in white: no data available. ◌: <10 MU/g tissue; ●: 10–100 MU/g tissue; ◎: 100–1000 MU/g tissue; ✖: > 1000 MU/g tissue; -: no data available. 1 MU = the amount of TTX that kills a male mouse of 20 g in 30 min after intraperitoneal administration (about 0.2 μg of TTX).

			Muscle	Skin	Testis	Ovary	Liver	Intestine	Blood
**Diodontidae**	***Chilomycterus***	*affinis*	◌	◌	-	◌	◌	◌	-
		***reticulatus***	-	-	-	-	-	-	-
	***Cyclichthys***	***orbicularis***	-	-	-	-	-	-	-
		***spilostylus***	-	-	-	-	-	-	-
	***Diodon***	***eydouxii***	-	-	-	-	-	-	-
		***holocanthus***	◌	◌	-	◌	◌	◌	-
		***hystrix***	◌	◌	-	◌	◌	◌	-
		***liturosus***	-	-	-	-	-	-	-
**Tetraodontidae**	***Arothron***	***hispidus***	●	✖	◌	◎	◎	◎	-
		*firmamentum*	◌	●	◌	◎	◌	◌	-
		***manilensis***	●	◎	-	◎	●	●	-
		***mappa***	●	●	-	◎	●	●	-
		***nigropunctatus***	◎	◎	-	◎	◎	◎	-
		***stellatus***	●	●	✖	✖	●	●	-
	***Canthigaster***	***rivulata***	◌	◎	-	◌	●	●	-
	***Chelonodon***	***patoca***	◎	✖	◎	◎	◎	✖	-
	***Lagocephalus***	*gloveri*	◌	◌	◌	◌	◌	◌	-
		***inermis***	◌	◌	◌	◌	◎	◌	-
		***lunaris***	◉	◎	◌	◌	◌	◌	-
		*wheeleri*	◌	◌	◌	◌	◌	◌	-
		***sceleratus***	●	●	-	✖	●	◎	-
	***Takifugu***	*chinensis*	-	-	-	✖	✖	-	-
		*chrysops*	◌	◎	◌	◎	◎	●	◌
		*exascurus*	◌	◎	◌	✖	◎	-	-
		*flavidus*	●	◎	◎	✖	✖	◎	-
		*niphobles*	●	◎	●	✖	✖	✖	-
		*obscurus*	◌	◎	◌	✖	◎	◎	-
		*pardalis*	◌	◎	●	✖	✖	◎	◌
		*poecilonotus*	●	◎	◎	✖	✖	◎	-
		*porphyreus*	◌	◎	◌	✖	✖	◎	-
		*pseudommus*	◌	●	◌	✖	●	●	-
		***rubripes***	◌	◌	◌	✖	◎	●	◌
		*snyderi*	●	◎	◌	✖	✖	◎	-
		*stictonotus*	◌	●	◌	◎	◎	◌	-
		*vermicularis*	◌	◎	◌	◎	◎	●	-
		*xanthopterus*	◌	◌	◌	◎	◎	●	-
	***Sphoeroides***	***pachygaster***	◌	◌	◌	◌	◌	◌	-
	*Tetraodon*	*alboreticulatus*	●	●	-	✖	●	◎	-
		*nigrovridis*	●	◎	-	-	◌	●	-
		*steindachneri*	◌	◌	-	-	◌	◌	-

## Data Availability

The datasets analyzed for this study can be found in this paper.

## Supplementary Materials

The following supporting information can be downloaded at https://www.mdpi.com/article/10.3390/ani13132113/s1: Table S1: NISP, MNE, MNI and burning marks of Diodontidae at Bubog I.; Table S2: NISP, MNE, MNI and burning marks of Diodontidae at Bubog II; Table S3: NISP, MNE, MNI and burning marks of Diodontidae at Bilat Cave.
