# Supplementary material for: The Exploitation of Toxic Fish from the Terminal Pleistocene in Maritime Southeast Asia: A Case Study from the Mindoro Archaeological Sites, Philippines
Source: Animals (Basel). 2023 Jun 26;13(13):2113. doi: 10.3390/ani13132113 (PMC10339915; doi:10.3390/ani13132113)
Supplement: Supplementary file 1 [file animals-13-02113-s001.zip › animals-2440604-supplementary.pdf]

animals

MDPI

Supplementary material for

The Exploitation of Toxic Fish from the Terminal Pleistocene in Maritime Southeast Asia: A Case Study from the Mindoro Archaeological Sites, Philippines

Clara Boulanger [1,2,3,*], Alfred Pawlik [4,5,6], Sue O'Connor [2,7], Anne-Marie Sémah [1], Marian C. Reyes [8,9] and Thomas Ingicco [1]

1 UMR 7194 Histoire Naturelle de l'Homme Préhistorique, Muséum National d'Histoire Naturelle, 75005 Paris, France
2 Archaeology and Natural History, School of Culture, History and Language, College of Asia and the Pacific, Australian National University, Canberra, ACT 2601, Australia
3 Japan Society for the Promotion of Science International Research Fellow, Department of Modern Society and Civilization, National Museum of Ethnology, Osaka 565-8511, Japan
4 Department of Sociology and Anthropology, School of Social Sciences, Ateneo de Manila University, Quezon City 1108, Philippines
5 TRACES ASIA, 3F Eduardo J. Aboitiz Sandbox Zone, Areté, Ateneo de Manila University, Quezon City 1108, Philippines
6 Department of Early Prehistory and Quaternary Ecology, Eberhard Karls Universität Tübingen, Schloss Hohentübingen, 72074 Tübingen, Germany
7 ARC Centre of Excellence for Australian Biodiversity and Heritage, Australian National University, Canberra, ACT 2601, Australia
8 The National Museum of the Philippines, Manila 1000, Philippines
9 School of Archaeology, University of the Philippines Diliman, Quezon City 1101, Philippines
* Correspondence: clara.boulanger@mnhn.fr

Supplementary Table S1. NISP, MNE, MNI and burning marks of Diodontidae at Bubog I.

Datings	Layer	NISP to family level	NISP to species level	Anatomical element	MNI	Burning
Neolithic	1	4	1	Dentary (MNE = 1)	1	0
				Dermal spine (MNE = 3)		3
	2	5	0	Dermal spine (MNE = 5)	1	5
4,848-4,651 cal. BP	3	9	0	Dermal spine (MNE = 9)	1	1
	4	9	0	Dermal spine (MNE = 9)	1	7
5,628-5,309 cal. BP	5	41	1	Premaxilla (MNE = 1)	1	1
				Unidentified jaw fragment (MNE = 1)		1
				Dermal spine (MNE = 39)		35
	6	68	0	Dermal spine (MNE = 68)	1	66
	7	7	0	Dermal spine (MNE = 7)	1	7
7,341-7,012 cal. BP	8	5	0	Dermal spine (MNE = 5)	1	4
33,040-31,736 cal. BP	9	0	0	NA	0	0
	10	0	0	NA	0	0
	11	0	0	NA	0	0
	12	0	0	NA	0	0
**Total**	**x**	**148**	**2**	**NA**	**8**	**130**

Supplementary Table S2. NISP, MNE, MNI and burning marks of Diodontidae at Bubog II

Datings	Layer	NISP to family level	NISP to species level	Anatomical element	MNI	Burning
Neolithic/Pre-colonial period	1	0	0	NA	0	0
	2	1	0	Dermal spine (MNE = 1)	1	0
	3	1	0	Dermal spine (MNE = 1)	1	0
	4	6	0	Dermal spine (MNE = 6)	1	0
c. 4,800-4,600 cal. BP	5	6	1	Premaxilla (MNE = 1)	1	0
			2	Dermal spine (MNE = 5)		2
	6	2	0	Dermal spine (MNE = 2)	1	0
5,930-5,744 cal. BP	7	8	1	Dentary (MNE = 1)	1	0
				Dermal spine (MNE = 7)	1	2
	8	5	0	Dermal spine (MNE = 5)	1	0
	9	1	0	Dermal spine (MNE = 1)	1	0
	10	0	0	NA	0	0
	11	0	0	NA	0	0
9,536-9,316 cal. BP	12	0	0	NA	0	0
**Total**		**30**	**4**		**9**	**4**

Supplementary Table S3. NISP, MNE, MNI and burning marks at Bilat Cave

Datings	Layer	NISP to family level	NISP to species level	Anatomical element	MNI	Burning
8,185-8,033 cal. BP (bottom)	1	2	0	Dermal spine (MNE = 2)	1	0
	2	11	2	Premaxilla (MNE = 1)	1	0
				Dentary (MNE = 1)		0
				Dermal spine (MNE = 9)		9
13,790-13,596 cal. BP	3	1	0	Dentary (MNE = 1)	1	1
	4	1	0	Dermal spine (MNE = 1)	1	1
**Total**		**15**	**2**		**4**	**11**
